# Australian Scorpion *Hormurus waigiensis* Venom Fractions Show Broad Bioactivity through Modulation of Bio-Impedance and Cytosolic Calcium

**DOI:** 10.3390/biom10040617

**Published:** 2020-04-16

**Authors:** David M. Housley, Jeremy L. Pinyon, Georg von Jonquieres, Chamini J. Perera, Michael Smout, Michael J. Liddell, Ernest A. Jennings, David Wilson, Gary D. Housley

**Affiliations:** 1 Translational Neuroscience Facility and Department of Physiology, School of Medical Sciences, UNSW Sydney, Sydney, NSW 2052, Australia; david.housley@my.jcu.edu.au (D.M.H.); j.pinyon@unsw.edu.au (J.L.P.); g.jonquieres@unsw.edu.au (G.v.J.); h.c.perera@unsw.edu.au (C.J.P.); 2 Department of Otolaryngology, Sunshine Coast University Hospital, Sunshine Coast, QLD 4575, Australia; 3 College of Medicine and Dentistry, Cairns Campus, James Cook University, Cairns, QLD 4878, Australia; ernest.jennings@jcu.edu.au; 4 Australian Institute of Tropical Health and Medicine, James Cook University, Cairns, QLD 4878, Australia; michael.smout@jcu.edu.au (M.S.); david.wilson4@jcu.edu.au (D.W.); 5 Centre for Molecular Therapeutics, James Cook University, Cairns, QLD 4878, Australia; 6 Centre for Tropical Environmental and Sustainability Science, College of Science & Engineering, Cairns Campus, James Cook University, Cairns, QLD 4878, Australia; michael.liddell@jcu.edu.au

**Keywords:** membrane biophysics, scorpion toxins, xCELLigence Real Time Cell Analysis, GCaMP5G calcium reporter, ryanodine receptors, calcium store, HEK293 cells, scorpion envenomation, recombinant rabbit RyR1, Ca^2+^ biosensor

## Abstract

Scorpion venoms are a rich source of bioactive molecules, but characterisation of toxin peptides affecting cytosolic Ca^2+^, central to cell signalling and cell death, is limited. We undertook a functional screening of the venom of the Australian scorpion *Hormurus waigiensis* to determine the breadth of Ca^2+^ mobilisation. A human embryonic kidney (HEK293) cell line stably expressing the genetically encoded Ca^2+^ reporter GCaMP5G and the rabbit type 1 ryanodine receptor (RyR1) was developed as a biosensor. Size-exclusion Fast Protein Liquid Chromatography separated the venom into 53 fractions, constituting 12 chromatographic peaks. Liquid chromatography mass spectroscopy identified 182 distinct molecules with 3 to 63 components per peak. The molecular weights varied from 258 Da—13.6 kDa, with 53% under 1 kDa. The majority of the venom chromatographic peaks (tested as six venom pools) were found to reversibly modulate cell monolayer bioimpedance, detected using the xCELLigence platform (ACEA Biosciences). Confocal Ca^2+^ imaging showed 9/14 peak samples, with molecules spanning the molecular size range, increased cytosolic Ca^2+^ mobilization. *H. waigiensis* venom Ca^2+^ activity was correlated with changes in bio-impedance, reflecting multi-modal toxin actions on cell physiology across the venom proteome.

## 1. Introduction

Scorpion venom typically represents a rich resource for drug discovery as it contains a complex mixture of small peptides, proteins (enzymes, phospholipases, and proteases), amino acids, biogenic amines, lipids, carbohydrates, and inorganic salts [1,2,3]. The physiological effects of the venoms are primarily mediated by scorpion toxin (SCTX) peptide actions on various ion channels present within excitable cell membranes; diversity that developed over millennia in response to extended positive selection pressure via predator–prey interactions [4,5,6]. These peptides are thus able to modulate central and peripheral nervous system excitability, alter smooth and skeletal muscle activity, and cause membrane destabilization. Scorpion envenomation causes significant morbidity and mortality in many tropical and subtropical countries, with over 1 million stings and ~2600 deaths annually, the majority occurring in children [7]. Stings by the Moroccan *Androctonus mauretanicus mauretanicus* scorpion for example, have a 10% fatality rate in children [8]. Envenomation with these components often results in rapid local hyperalgesia and inflammation, followed by paresthesia and altered sensation lasting for hours to days. Systemic effects are seen in approximately 10% of cases, and include symptoms such as nausea, vomiting, lethargy, dysrhythmias, and seizures [9]. 

SCTX peptides can be categorised by the size of their peptide chain, either short chain (usually K^+^ channel blockers), or long chain peptides (often Na^+^ channel modulators) [10,11], or generically as either disulfide bridge-containing peptides or non-disulfide bridge-containing peptides [12]. The vast majority of research conducted to date has focused on SCTX peptide action on voltage-gated Na^+^ (Nav) channels [13,14,15,16,17], and voltage-gated K^+^ (Kv) ion channels [8,18,19,20,21], with Ca^2+^ channels [22,23] and Cl^-^ channels relatively neglected [24]. This mirrors the typical relative abundance of toxin components against K^+^ and Na^+^ channels (for example ~38% each in the red Indian scorpion *Mesobuthus tamulus*, compared to 0.8% for a Ca^2+^ channel toxin [3]). Identification of SCTXs with actions on Ca^2+^ entry channels and Ca^2+^ store release would offer significant potential for development of therapeutic agents and molecular carriers [25,26,27] and it is unclear what the breadth of impact on Ca^2+^ mobilization is across the highly diverse molecular mosaic of scorpion venoms. 

The dynamics of cytosolic Ca^2+^ mobilization and membrane conductance are broadly interwoven. Activation of several classes of Ca^2+^ permeable plasma membrane ion channels can affect membrane conductance directly, as well as indirectly, via activation of Ca^2+^-dependent K^+^ channels and Ca^2+^ store activation (Ca^2+^ ‒ induced Ca^2+^ ‒ release, CICR). Reciprocally, activation of ryanodine receptor (RyR)-gated Ca^2+^ stores and inositol trisphosphate (IP_3_) receptor-gated Ca^2+^ stores in the endoplasmic and sarcoplasmic reticulum can elicit ‘capacitive Ca^2+^ entry’ [28,29]. CICR is strongly tied to RyR1-3, large macromolecular ion channel complexes which derive their name from the agonist action of the plant alkaloid ryanodine, and are notable for their activation by caffeine [30]. RyRs are also activated by allosteric coupling to the L-type Ca^2+^ channel, and by modulation through IP_3_R [29,31,32,33]. RyRs have widespread tissue distribution, with RyR1 found in skeletal muscle and in cerebellar Purkinje cells [34], RyR2 within cardiac muscle, brain, visceral, and arterial smooth muscle [35], and RyR3 in smooth muscle, the diaphragm, epithelial cells, and the brain [36,37]. RyR mutations underlie disease states such as polymorphic ventricular tachycardia and malignant hypertension [38,39]. A small number of SCTX peptides, termed ‘calcins’, have recently been identified to rapidly penetrate the cell membrane and bind with high affinity to RyR channels [40,41]. Upon binding they induce long-lived channel subconductance states, thereby modulating cytosolic Ca^2+^ [25,41,42,43], which acts as a second-messenger, increasing cellular excitability, neurotransmitter release, gene expression, and intracellular metabolism [44]. These calcins are also able to carry impermeable molecules across the membrane with them, making them potentially valuable small molecule tools for intracellular drug delivery [45].

The SCTX φ-LITX-Lw1a from the Australian scorpion *Hormurus waigiensis* (*Liocheles waigiensis*) [46] is the most potent identified calcin-like peptide, modulating RyR1 and RyR2 at femtomolar concentrations (AC_50_ of 6.0 pM and 2.0 pM, respectively) [47]. This is the only SCTX peptide that has been characterised from the whole venom of *H. waigiensis* to date. The venom comprises a large pool of uncharacterised peptides [47], which therefore represents a considerable source of potential ion channel modulators, raising the question investigated here as to the breadth of bioactivity of these molecules on cell calcium dynamics and associated membrane conductance. We sought to determine the extent of cytosolic Ca^2+^ mobilization and impact on bio-impedance by *H. waigiensis* scorpion venom fractions delineated by size exclusion fast protein liquid chromatography (SE-FPLC) and characterised by liquid chromatography mass spectroscopy (LCMS). To this end, we established a highly sensitive bioreporter HEK293 cell line that stably expressed the genetically encoded Ca^2+^ reporter GCaMP5G and the type 1 ryanodine receptor (RyR1) (rHEK293).

## 2. Materials and Methods 

### 2.1. Generation of the Recombinant HEK293-RyR1-GCaMP5G Cell Line

Untransfected human embryonic kidney 293 (HEK293) cells have been reported to have varying capacity for gating of Ca^2+^ stores via the endogenous RyR expression, as evident from differences in sensitivity to caffeine [29,48,49]. This contrasts to the robust IP_3_R-gated Ca^2+^ signalling in these cells, as evident in many studies where the M3 muscarinic receptor agonist carbachol has been employed to drive Ca^2+^ store release [49]. To achieve a bioreporter cell line with optimum sensitivity to both classes of Ca^2+^ stores, alongside Ca^2+^ entry, a custom-developed recombinant human embryonic kidney 293 (rHEK293) cell line stably over-expressing the rabbit RyR1 calcium-store channel (International Nucleotide Sequence Database Collaboration number: NM_001101718, GenBank, provided by Dr. Paul Allen, University of California Davis, and Angela Dulhunty, Australian National University), and the GCaMP5G genetically encoded Ca^2+^ reporter (gift from Douglas Kim and Loren Looger; Addgene plasmid no.31788) [50] was developed. The details of the development and validation of this cell line are described in the Supplementary Materials and associated Figure S1.

### 2.2. Scorpion Venom Collection

**Figure 1:**
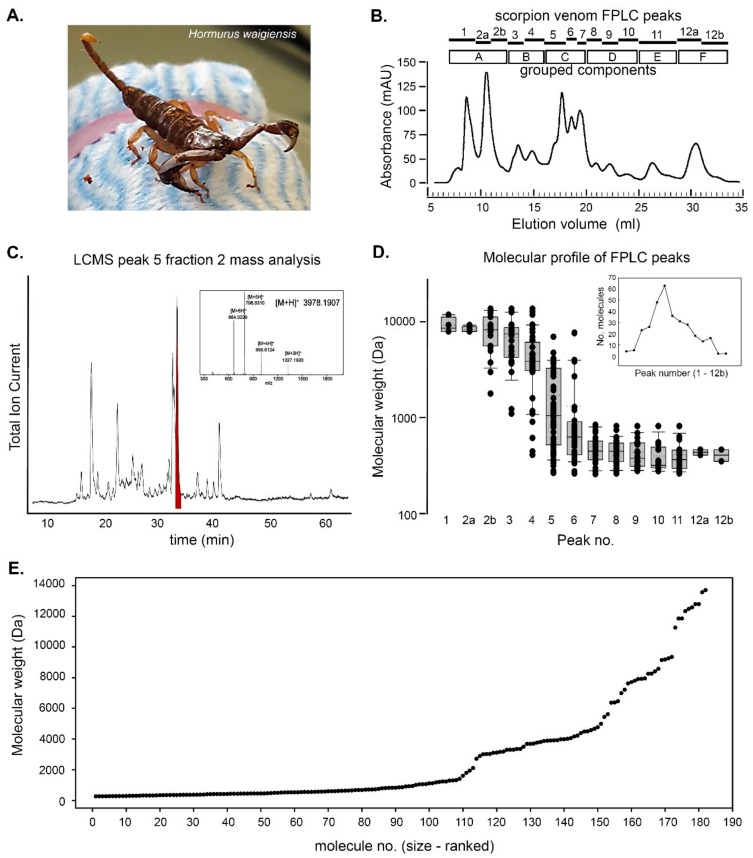
Molecular analysis of pooled venom from Australian scorpion *Hormurus waigiensis.* (**A**) Image of a scorpion on the pad used for venom collection by electrical stimulation of the telson. (**B**) The crude venom chromatogram at A_280nm_ from SE-FPLC highlighting the 12 resolved peaks (designated as peak samples 1–12b); venom Pools (A–F) used for the bio-impedance study are shown in relation to the corresponding peak samples and the elution fractions. (**C**) An example of the LCMS analysis for the second elution fraction (EF 20) within peak sample 5, where 41 molecular weights were identified. The inset mass spectrum illustrates an example of the underlying mass spectra of the highlighted (red) LCMS chromatogram peak and the corresponding molecular weight of the molecule. (**D**) A box plot analysis of the molecular weight distributions of all 14 peak samples (see also Table S1). The boundaries of the box plots represent 25% and 75% percentiles, the solid line is the median, the dashed line is the mean, 95% percentiles are also shown; individual data points are overlaid. The inset shows the number of distinct molecules identified within each peak sample. (**E**) Size-ranked plot of the molecular weights of the 182 molecules identified in the venom fractions.

This study used the venom milked from *Hormurus waigiensis,* a rainforest scorpion with widespread distribution across South-East Asia, the Pacific, and throughout the Australian east coast [51] (Figure 1A). These scorpions were collected from a rainforest environment, Cairns, Queensland, Australia, from November 2015 to April 2016. Scorpions were then individually held in 170 × 110 × 50 mm clear plastic containers, each containing moist autoclaved soil and a rock. The containers were kept in incubators (Wisecube WGC-450) at 28 °C on a 14/10 light and dark cycle. The scorpions were regularly fed crickets, relative humidity was maintained at 70%, and the containers were changed at 3-week intervals. 

Scorpion venom was collected via electrostimulation. The scorpions were initially secured to a block of foam covered with cloth material (Figure 1A) using rubber bands. A pair of electrodes moistened with saline were then placed into contact with opposing sides of the scorpion telson, and a square wave stimulator (Arthur H. Thomas Co. model Z789) was used to apply positive monophasic 20 V stimuli (5.5 pulses/sec, duration 15 ms, continuous). The amplitude of the pulses was progressively increased towards 30 V until venom was exuded. The venom was captured by positioning a micropipette tip over the scorpion aculeus, and was then stored at −80 °C.

### 2.3. Scorpion Venom Analysis

To obtain the venom fractions, stored whole venom was pooled from across the scorpion population (14 animals, ~50 µL) and then diluted with 150 μl of degassed phosphate buffered saline (PBS), centrifuged for 10 min, and filtered through a 0.22 μm Millipore filter. Venom profiles were subsequently obtained by SE-FPLC (ÄKTA™, GE Healthcare) using a Superdex 75 10/300 (Tricorn) GL Column (13 μm, 10 × 300 mm) with PBS as the carrier (500 µL/minute for 45 mL). Venom component elution was monitored at an absorbance wavelength of 280 nm. Fractions (500 µL) were collected into a 96 well-plate and stored at 4 °C (~530-fold dilution). Venom profiles were then processed and exported using UNICORN 5.20 software (2008, GE Healthcare). Individual elution fractions (EF) obtained from SE-FPLC were then analysed by LCMS using a Shimadzu LCMS-2020 mass spectrometer coupled to a Shimadzu Prominence HPLC system (Shimadzu, Japan) to observe the composition and determine the molecular weights of the molecules present in each fraction. Fractions (20 µL) were injected via an autosampler (Shimadzu SIL-20AC-HT) onto a reversed-phase high-performance liquid chromatography column (Phenomonex Aeris PEPTIDE XB-C18, 3.6 µm, 100Å, 150 × 2.1 mm) at 30 °C. Solvent (buffer A: 0.1% formic acid/water; buffer B: 90% acetonitrile/0.09% formic acid/water) was delivered via Shimadzu LC-20AD pumps at a flow rate of 0.250 mL/min. Samples were washed on the column for 5 min to remove salt from the SE-FPLC PBS buffer, then eluted with a 1% gradient (0–60% buffer B, 60 min; 60–90% buffer B, 5 min; 90% buffer B, 5 min; 90–0% buffer B, 5 min; 0% buffer B, 10 min), and the UV absorbance observed at 214 nm and 280 nm on a Shimadzu SPD-20A detector. Mass spectra were collected in positive ion mode over a scan range of m/z 250–2000 with a detector voltage of 1.15 kV, nebulizing gas flow of 1.5 L/min, and drying gas flow of 3.0 L/min. Data were collected and analysed using the Shimadzu LabSolutions v5.80 software.

### 2.4. Measurement of rHEK293-RyR1-GCaMP5G Cell Monolayer Bioimpedance 

A high-throughput screen to investigate the biophysical actions of *H. waigiensis* venom on the rHEK293 biosensor cells was undertaken using the xCELLigence RTCA Single Plate system (ACEA Biosciences, Inc.), which provided real-time bio-impedance measurements. This cellular impedance assay is reported as the unit-less “Cell Index” (CI). CI reflects a broad range of cell responses that affect electric current flow through the cell layer overlying an array of gold microelectrodes, including effects on cell shape and number, cell-cell junctions and cell–substrate adhesion, ion channel activation, and G protein-coupled receptor (GPCR)-mediated cytosolic Ca^2+^ elevation [52]. The 96 well E-plate (ACEA Biosciences, Inc.) was initially loaded with the trypsinised rHEK293 cells (150 µL Dulbecco’s modified Eagle’s medium (DMEM) with ~5000 cells per well), placed within the system sensor inside the cell incubator, and then allowed to settle overnight. On the following day, measurement of cell index was recorded just prior to the application of *H. waigiensis* venom. Venom samples (50 µL at 1: 2,100 final dilution) were then applied to all wells via a multi-tip pipette. Combining of the elution fractions into six venom Pools (A–F) was necessary to provide sufficient material for replication across multiple wells. The elution fractions comprising these sample pools were: Pool A (peak samples 1–2a, b; EF 1‒11); Pool B (peak samples 3–4; EF 12‒18); Pool C (peak samples 5–7; EF 19‒26); Pool D (peak samples 8–10; EF 27‒36); Pool E (peak sample 11; EF 37‒43); Pool F (peak samples 12a, b; EF 44‒53). Recording commenced at 15 sec intervals (for a total of 1500 s). Data were analysed using RTCA software V.2 (ACEA Biosciences, Inc., 2013). 

Data are presented as mean normalised CI ± s.e.m, with CI = 1 representing the CI measurement just prior to toxin addition. GraphPad Prism (v. 7) was used to produce the graphs. SigmaPlot (Systat v. 12.5) was used for statistical analysis. This included individual one-sample *t*-tests (two-tailed) at 2.5, 5, and 25 min time points on normalized background subtracted data to assess a difference from a predicted mean of 0. Where data were not normally distributed, as determined by Systat, a one sample Signed Rank Test was undertaken. The alpha value for significance was 0.05. Two-way repeated measure analysis of variance (ANOVA), with post-hoc Holm–Sidak multiple pairwise comparison of pooled venom components was performed for runs 10 (2.5 min), 20 (5 min), and 100 (25 min).

### 2.5. Calcium Imaging

The stably transfected rHEK293 cell line was plated onto glass coverslips. At 80% confluence, the cells were placed into an imaging bath (0.5 mL DMEM media) which was maintained by a pump system (Gilson minipuls 3) at a constant flow rate (360 µL/min). *H. waigiensis* venom was superfused into the imaging bath containing rHEK293 cells at a final dilution of ~1: 10,000 (in 3 mL) for 500 s. This was followed by a 1000 second DMEM media washout period, 500 s of 4 mM caffeine exposure (positive control), and then a 1000 s media washout. This protocol was conducted for raw whole *H. waigiensis* venom (three repeats), PBS elutant control from the SE-FPLC process (three repeats), media-only control (two repeats), and in triplicate for 14 peak samples reconstituted from the 53 elution fractions (EF) comprising the 12 chromatographic SE-FPLC peaks identified (Figure 1B): Peak sample 1 (EF 1–5); peak sample 2a (EF 6–8); peak sample 2b (EF 9–11); peak sample 3 (EF 12–14); peak sample 4 (EF 15–18); peak sample 5 (EF 19–22); Peak sample 6 (EF 23,24); peak sample 7 (EF 25,26); peak sample 8 (EF 27–29); peak sample 9 (EF 30–32); peak sample 10 (EF 33‒36); peak sample 11 (EF 37–43); peak sample 12a (EF 44–48); peak sample 12b (EF 49–53).

Calcium imaging was conducted using a Zeiss 710 Laser Scanning Microscope with a W N-Achroplan 10×/0.3 W (DIC) M27 water immersion objective at 1.5x zoom; 488 nm excitation/542 nm emission, 10 s sampling rate with no averaging, using Zeiss Zen Blue 2012 software. Images (512 × 512 pixels; 1.11 µm/pixel, 8 bit) were generated for each time point. Image analysis was undertaken using Image J software (1.50i, National Institutes of Health USA) which calculated mean pixel intensity for the whole of field region of interest. Increases in cytosolic Ca^2+^ following venom and caffeine application were assessed as changes in (F/F_0_), where F_0_ is the average of three whole field mean pixel intensity values (covering 30 sec) immediately prior to changing to the test solution, and F is the average of three whole field mean pixel intensity values immediately prior to media washout.

Sigmaplot (Systat v.12.5) as used for statistical analysis. Data are presented as mean ± s.e.m. For whole venom, SE-FPLC peak samples, controls (PBS and media samples) and caffeine, Ca^2+^ responses were determined using single sample *t*-tests (single-tailed), or one sample Signed Rank Tests if data were not normally distributed. The alpha value for significance was 0.05, assessing a difference from a predicted mean value of 1.00. With validation of normal distribution, comparison of samples against the control group was then undertaken using one-way ANOVA, with post-hoc Bonferroni-corrected pairwise comparison. *T*-tests (two-tailed) were used to compare the SCTX Ca^2+^ responses against the caffeine responses.

## 3. Results

### 3.1. Scorpion Venom Molecular Characterisation

Pooled whole *H. waigiensis* venom from 14 animals (Figure 1A) in phosphate buffered saline (PBS) carrier was fractionated by SE-FPLC into 53 × 500 µL fractions across 12 chromatographic peaks (2–7 fractions per peak). The study functionally characterised these as 14 peak samples (designated: 1, 2a, 2b, 3–12a, 12b) as shown in Figure 1B. The molecular diversity within each peak was assessed using LCMS, characterizing the composition and molecular weight of the molecules present (Figure 1C–E). For example, Peak 5 comprised four fractions, with LCMS identifying 41 different molecules within the second of the four fractions (Figure 1C). Peak 5 had the greatest molecular diversity across all the peaks, with 63 different molecules ranging in molecular weight from 265 Da to 6975 Da (Figure 1D). The molecular weights of the molecules in the first three peaks were all above 1.7 kDa, consistent with the size of K^+^ and Na^+^ channel-targeting toxins [3]. The smallest molecule in Peak 4 was 409 Da, while Peaks 5–11 had molecules of around 260 Da; Peak 12 (combining peak samples 12a and 12b) consisted of just three small molecules of 351, 408, and 465 Da. The least diversity in high molecular weight molecules was seen in Peak 1 and Peak 2a, (four molecules 7.9–11.8 kDa and five molecules 7.9–9.3 kDa, respectively). Molecules of equivalent molecular weights to a decimal point across adjacent eluction fractions were considered to be identical, being within the margin of error for the MS mass determination (Figure 1D). Overall, 182 distinct molecules were identified from the venom, ranging in size from 258 Da to 13695 Da. The majority of molecules were less than 1 kDa (53%) and the molecular size distribution exhibited a logarithmic function with increasing complexity of the molecules above an inflection point at 1.3 kDa (Figure 1E). The molecular weights for individual molecules isolated within each peak are provided as Supplementary Materials Table S1; the list of molecular weights of the molecules discriminated across the fractions are provided as Supplementary Materials Table S2. 

### 3.2. Venom-Induced Membrane Impedance Responses

**Figure 2:**
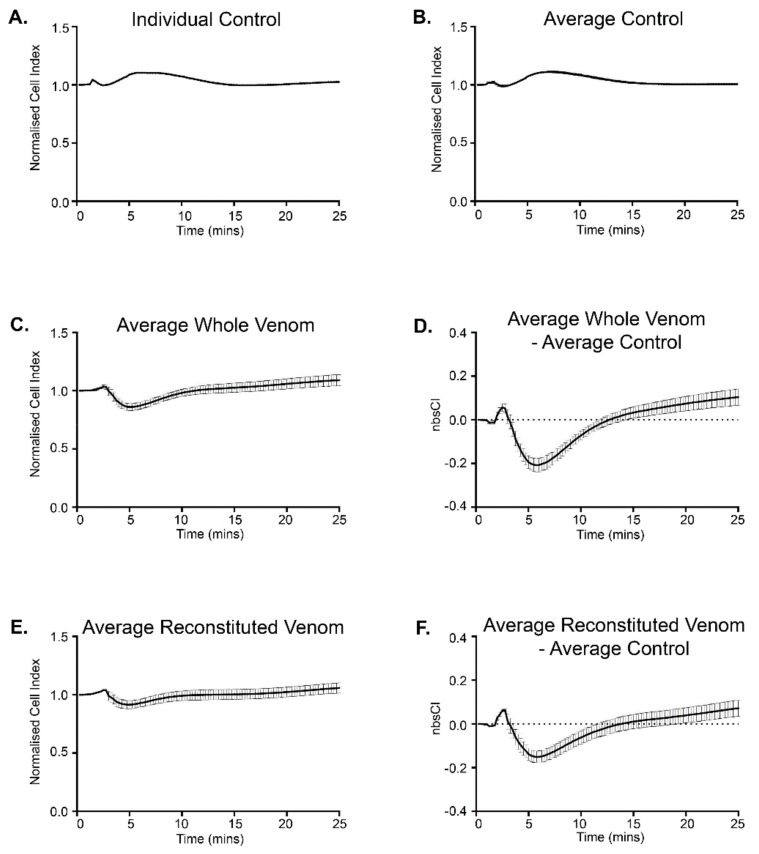
xCELLigence data showing changes in GCaMP5G-RyR1-HEK293 monolayer impedance (normalised cell index (CI)) in response to application of control, whole venom or reconstituted venom from the Australian scorpion *Hormurus waigiensis*. (**A**) The effect of adding a sample of the background control (SE-FPLC phosphate buffered saline) to a single well containing rHEK293 cells reflects the sensitivity to fluid changes. (**B**) Average of the background control responses. (**C**) Average response following application of whole venom. (**D**) Average whole venom response corrected for the background (average whole venom—average control). (**E**) Average response of the reconstituted scorpion venom. (**F**) Corrected response for reconstituted venom (average reconstituted venom—average control). For (**D**) and (**F**), nbsCI = average normalized background subtracted CI. Plots (**B**)–(**F**) show mean ± s.e.m.; error bars in (**B**) are not evident due to the minimal variance (*n* = 8); *n* = 6 for each condition.

Investigation of the effect of whole and fractionated venom on membrane impedance was undertaken using the xCELLigence platform. Negative controls consisting of PBS eluted from the SE-FPLC were used to establish a baseline profile with solution switching (*n* = 8). HEK293 cells are known to be sensitive to mechanical disturbances, as exhibited by changes in cytosolic Ca^2+^ [49]. This responsivity was confirmed in the present study by the readout of the GCaMP5G-RyR1-HEK293 (rHEK293) cell monolayer impedance in these negative controls (Figure 2A,B). The profile was highly reproducible as evident in Figure 2B, which shows the average of the individual control responses. These data show a small transient increase in normalized cell index (CI) to 1.023 ± 0.011 (*n* = 5) at approximately 105 s, followed by a subsequent delayed increase in impedance to a maximum CI of 1.112 ± 0.009 at 7.5 min, where normalized CI = 1 prior to sample addition. This average control profile was subsequently subtracted from the individual responses to whole venom (*n* = 6), reconstituted whole venom (*n* = 6), and pooled SE-FPLC elution fractions: Pool A (*n* = 8); Pool B (*n* = 9); Pool C (*n* = 7); Pool D (*n* = 5); Pool E (*n* = 7); Pool F (*n* = 6).

Whole venom (1:2100 dilution) elicited a clear triphasic bio-impedance response from the rHEK293 cell monolayers (Figure 2C), that was resolved by subtraction of the average control profile (Figure 2D). Figure 2D clearly shows an initial rise in impedance, reaching a maximal average normalized background subtracted CI (nbsCI) of 0.057 ± 0.018 at 2.5 min. The second part of the response was a significant decrease in impedance, maximal at approximately 5 min, with an average nbsCI of −0.194 ± 0.029. This was followed by a late rebound in impedance, reaching a maximum nbsCI of 0.104 ± 0.037 at 25 min. Re-pooling of all venom fractions gave essentially the same result as whole venom, which was a test for retention of bioactivity (Figure 2E,F). Similar to whole venom, this reconstituted venom had an initial increase in impedance at 2.5 min, average nbsCI = 0.062 ± 0.008, a significant decrease by 5 min, nbsCI = ‒0.139 ± 0.028, and a late rise at 25 min, nbsCI = 0.072 ± 0.037.

**Figure 3:**
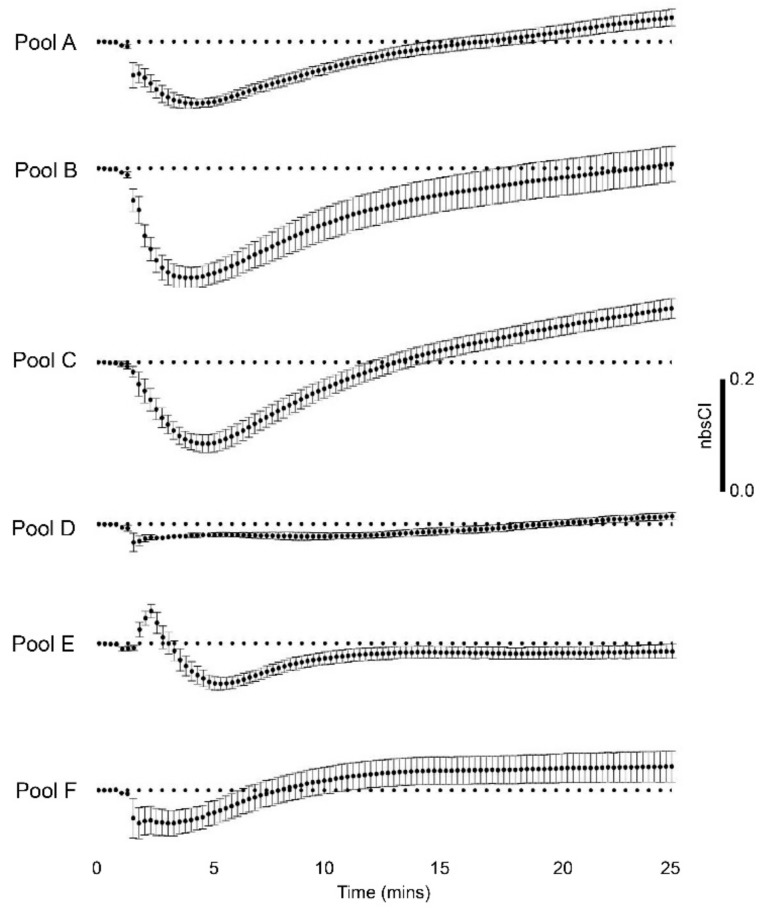
xCELLigence data showing average changes in GCaMP5G-RyR1-HEK293 cell monolayer impedance in response to application of venom components from Australian scorpion *Hormurus waigiensis*, corrected for average background control (nbsCI). Pooled venom sample A (peak samples 1, 2a, 2b); Pool B (peak samples 3, 4); Pool C (peak samples 5, 6, 7); Pool D (peak samples 8, 9, 10); Pool E (peak sample 11); Pool F (peak samples 12a, 12b).: SEM error bars shown (*n* = 5–9 replicates).

**Figure 4:**
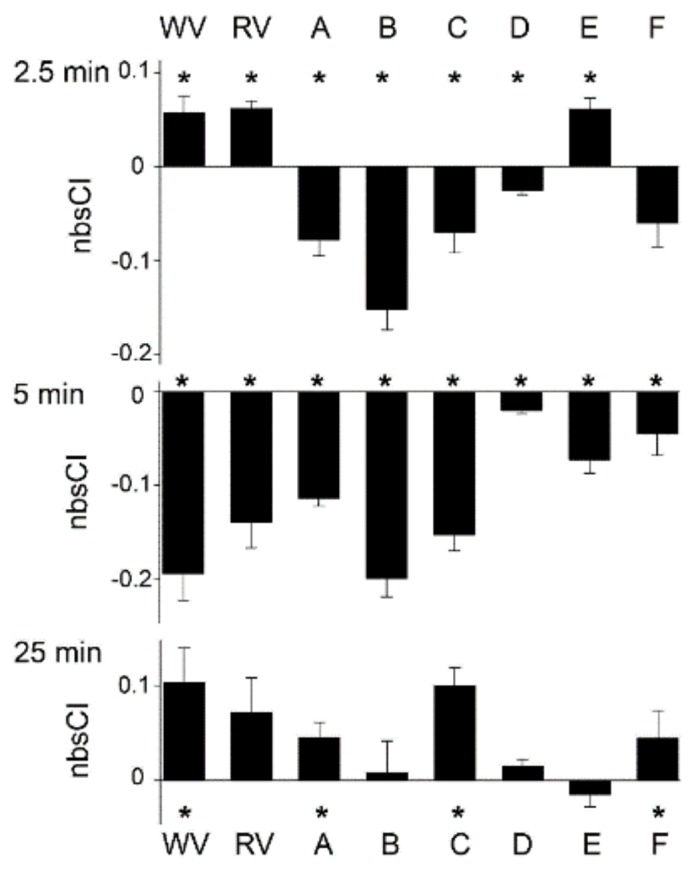
Summary of temporal profiles for xCELLigence—based bio-impedance to application of venom fractions from Australian scorpion *Hormurus waigiensis* to GCaMP5G-RyR1-HEK293 cell monolayers. Normalized background subtracted cell index data (nbsCI). WV, whole venom; RV, reconstituted venom; (**A**), venom Pool A (peak samples 1, 2a, 2b); (**B**), Pool B (peak samples 3, 4); (**C**), Pool C (peak samples 5, 6, 7); (**D**), Pool D (peak samples 8, 9, 10); (**E**), Pool E (peak sample 11); (**F**), Pool F (peak samples 12a, 12b)—see Supplementary Materials Tables S3–S6 for statistical comparisons. * indicates *p* < 0.05 for one sample *t*-tests; SEM error bars shown.

Figure 3 presents the xCELLigence responses of rHEK293 cells to pooled SE-FPLC fractions over 25 min (pooled samples as Pools A–F). Figure 4 and Supplementary Materials Table S3, summarize the temporal profiles for bioimpedance changes at 2.5, 5, and 25 minute census points for whole venom, reconstituted venom and the six fraction Pools. As seen in Figure 3, Pool E was the only venom pool that induced the initial increase in impedance seen with the whole venom at 2.5 min (Figure 2D), increasing the average nbsCI to 0.061 ± 0.013. Ergo the components contained within Pool E are likely to be solely responsible for the rapid initial increase in impedance. Pools A, B, and C were the main contributors to the subsequent decrease in impedance seen with whole venom at the 5 min mark (Figure 2D), with average nbsCIs of ‒0.114 ± 0.008, 1.994 ± 0.020, and ‒0.153 ± 0.017, respectively (Figure 3). Pool F also made a small contribution to this decrease at 5 min, with an average nbsCI of −0.045 ± 0.023 (Figure 3). The main contributor to the late increase in impedance seen with whole venom at 25 min (Figure 2D), was Pool C, with an average nbsCI of 0.101 ± 0.019 (Figure 3). Pools A and F made small contributions to this late increase as well, with average nbsCIs of 0.046 ± 0.016, and 0.045 ± 0.030 respectively (Figure 4A,F). Conversely, Pool D made no contribution to the bioactive profile seen with whole venom. The change in average nbsCI for Pool D was negligible across the key time points described above (2.5 min = −0.025 ± 0.005; 5 min = −0.020 ± 0.004; 25 min = 0.015 ± 0.007; Figure 3). Each venom sample pool demonstrated significant differences (*p* < 0.05) in their response for most census time points (2.5, 5, and 25 min) (Figure 4 and Supplementary Materials Table S3). Two-way repeated measure of analysis (ANOVA) revealed that the average nbsCI responses of the venom pools at 2.5, 5, and 25 min were significantly different from each other, overall *p* = 0.002, F = 4.82, DF = 5, as seen in Supplementary Materials Tables S4–S6. 

### 3.3. Venom-Induced Modulation of Cytosolic Calcium

**Figure 5:**
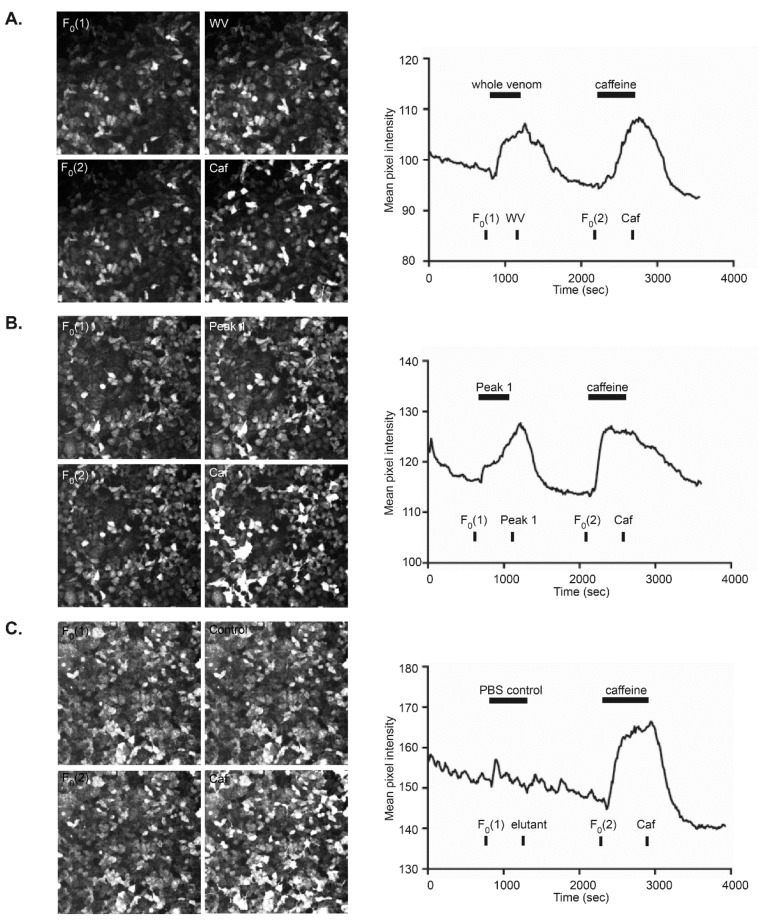
Examples of confocal fluorescence imaging of cytosolic Ca^2+^ in GCaMP5G-RyR1-HEK293 cells with application of whole venom (WV), peak sample 1 (Peak 1), caffeine and a PBS control. (**A**) Application of whole venom from Australian scorpion *Hormurus waigiensis* was compared to 4 mM caffeine positive control. (**B**) SE-FPLC peak sample 1 response compared to caffeine control. (**C**) PBS control compared to the caffeine positive control. The graphs reflect the continuous imaging (10 s sampling), with mean pixel intensity representing the whole-of-field (512 × 512 pixels). F_0_(1) for quantitative analysis comprised an average of 3 frames (30 s); the image shown is frame 2 of this sample, 20 s prior to addition of venom. Similarly, the WV image is the second of 3 frames used for analysis just prior to washout with media. F_0_(2) was used as the background reference just prior to addition of caffeine as a positive control.

Investigation of the capacity of whole and fractionated scorpion venom to modulate cytosolic Ca^2+^ was undertaken via confocal Ca^2+^ imaging using the rHEK293 cell line. Figure 5 shows examples of these experiments. Bath application of whole venom caused substantial increases in GCaMP5G-mediated fluorescence commencing within ~30 s, reflecting raised intracellular Ca^2+^ (Figure 5A). A comparable response profile was seen with subsequent application of caffeine (4 mM), following 1000 s of media washout. SE-FPLC peak sample 1 had a similar profile to that seen with whole venom, and the caffeine positive control (Figure 5B). Negative controls consisting of SE-FPLC PBS and DMEM media-only were used to establish a baseline response profile for potential biomechanical disturbance to the cells, which was minimal (Figure 5C). This experimental protocol was conducted in triplicate for whole venom and each of the 14 peak samples within the 12 SE-FPLC chromatographic peaks (Figure 1B). As described in the methods, F/F_0_ was calculated as F, the mean pixel intensity for the whole-of-field image (3 frames, 10 s sampling) following 470 s of the treatment,/F_0_, the mean pixel intensity obtained for the preceding reference period (average across all experiments = 116 ± 3 (*n* = 50)).

**Figure 6:**
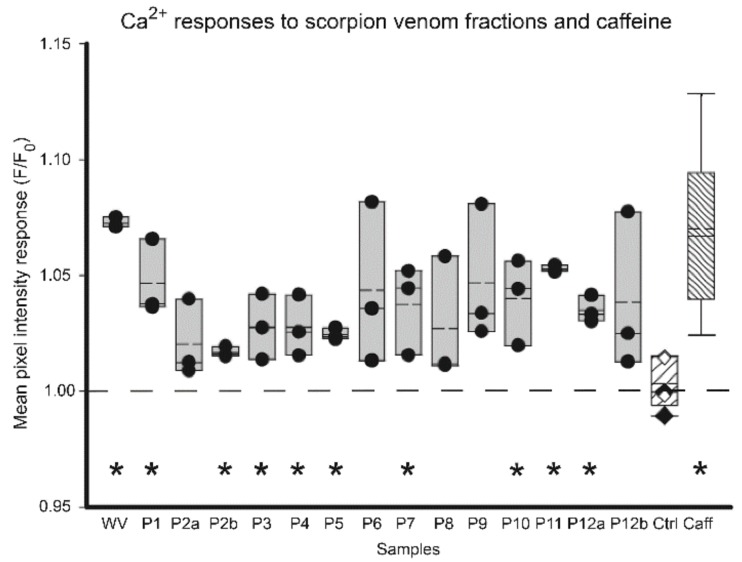
Summary of Ca^2+^ responses to Australian scorpion *Hormurus waigiensis* venom fractions and caffeine in GCaMP5G-RyR1-HEK293 cells. Boxplots with overlaid data for responses to whole scorpion venom (WV), 14 SE-FPLC peak samples (see Figure 1B), negative controls, and the 4 mM caffeine (Caff) positive control. Negative controls (Ctrl): PBS (black diamonds, n = 3) and DMEM media (white diamonds, n = 2) were combined. All venom tests were performed in triplicate. The caffeine data were combined from the 44 experiments. The box plots reflect 25% and 75% percentiles, with median shown as a solid line and mean shown as a dashed line; 95% percentile are shown as feather bars. * indicates a significant Ca^2+^ response (*p* < 0.05), as determined by one-sample *t*-tests (single-tailed).

Whole venom, 9 out of the 14 SE-FPLC peak samples (peak samples: 1, 2b, 3, 4, 5, 7, 10, 11, 12a), and caffeine, elicited statistically significant increases in cytosolic Ca^2+^, based on single sample t-tests (single-tailed), or one sample signed rank tests where data were not normally distributed (Figure 6, Supplementary Materials Table S7). For example, the average F/F_0_ for whole venom was 1.073 ± 0.001 (*p* = 0.00017; Table S7), this was reversible with washout and equivalent in magnitude to that seen with subsequent 4 mM caffeine applications (mean = 1.070 ± 0.006). The negative controls did not produce a significant Ca^2+^ response (mean = 1.003 ± 0.005, *p* = 0.268).

With validation of normal distribution, comparison of samples against the negative control group was then undertaken using one-way ANOVA revealing that overall, the application of SE-FPLC peaks to rHEK293 cells elicited significant Ca^2+^ responses (*p* = 0.001). Associated post-hoc Bonferroni-corrected pairwise comparison t-tests showed significant differences between whole venom, peak samples 1, 9, and 11, relative to the negative control group. Two-tailed *t*-tests were used to compare the Ca^2+^ response from application of whole venom and the nine significant peak samples (1, 2b, 3, 4, 5, 7, 10, 11, 12a) to that seen following 4 mM caffeine treatment. This showed that all these significant venom-induced responses were statistically indistinguishable from caffeine, except for Peak samples 2b and 5, that produced smaller Ca^2+^ responses (*p* = 0.0214 and *p* = 0.0470 respectively).

### 3.4. Comparison of Venom Activity Across Platforms

A rank order analysis of potency was performed using the 5-minute time point for the xCELLigence measurements against the 8 minute Ca^2+^ imaging time point to determine whether the potency of venom-induced biophysical responses identified by xCELLigence could be related to the magnitude of the venom-induced Ca^2+^ response. The Ca^2+^ responses were derived by summing the individual SE-FPLC peak responses that matched to the venom pools in the xCELLigence study. This qualitative analysis (Table 1) showed some correlation between the magnitude of impedance changes across the rHEK293 cell monolayers, and the degree of GCaMP5G-based Ca^2+^ fluorescence, as 2 of the top 3 responses matched potency between platforms (venom Pool C = second; Pool A = third). However, the most potent Ca^2+^ response activator (venom Pool D equivalent) was the weakest bioimpedance modulator (sixth), indicating that activity of some of the scorpion venom bioactive molecules are highly selective for Ca^2+^ mobilization, with limited secondary action on cell membrane properties.

## 4. Discussion

The present study identified surprising molecular diversity of *H. waigiensis* venom components that modulated cell bio-impedance and raised cytosolic Ca^2+^. This broad functional screening of the effects of scorpion venom components was enabled by development of the GCaMP5G‒RyR‒HEK293 cell Ca^2+^ sensor bioreporter model. SE-FPLC delineated 12 primary chromatographic peaks, which LCMS resolved as 182 distinct molecules, with 3 to 63 molecular components per peak. The screening was achieved by pooling discrete groups of molecules based on size separation. While these molecules were not individually resolved, ~45% were in the 1.5–14 kDa range (Figure 1D, Tables S1 and S2), which likely represent a diverse mix of scorpion toxin peptides [3,53]. The most potent molecules for altering cell bio-impedance were within this range (venom Pools A–C), while molecules below 1 kDa (principally Pools D–F) were most effective at elevating cytosolic Ca^2+^ (against this trend, peak sample 1, in Pool A, comprising just 4 molecules 7921–11846 Da elicited a strong Ca^2+^ response (Figure 1B,D and Figure 6, Table 1, Table S1)). While 9/14 peak samples promoted Ca^2+^ increases, no components elicited reductions in baseline Ca^2+^ signals. This suggests that these small molecules may be particularly effective at activating Ca^2+^ stores, possibly due to facilitated transport into the endoplasmic reticulum. The exception to this is the likely presence of calcins, which have a higher molecular weight, but are associated with facilitated plasma membrane translocation [40,41].

The distinct triphasic bio-impedance response seen using the xCELLigence platform following application of whole venom (transient increase at 2.5 min, substantial decrease around 5 min, late rise at 25 min), is consistent with a complex and diverse set of scorpion toxin peptide actions on the rHEK293 cells. These responses were able to be reconstructed and attributed to various pools of venom fractions, with Pool E (peak sample 11; 262–817 Da range) for example, making the predominant contribution to the increased impedance at 2.5 min, further indicating that the SCTX molecules within these mixtures have differential actions. This is expected given the molecular diversity that we identified within the venom (Figure 1D). Against this, peak samples 12a and 12b, with just three molecules (350, 408, 465 Da), showed significant Ca^2+^ mobilization and a reduction in bioimpedance at 5 min (xCELLigence venom Pool F).

Changes in xCELLigence platform-measured impedance across the rHEK293 cell monolayer likely reflects venom-induced alterations of the cell membrane conductance properties, as well as possible effects on morphology and/or transient changes in paracellular conductivity relative to the E-plate microelectrode array. This platform is particularly sensitive to Ca^2+^-mediated changes in cell properties, including those elicited by activation of Ca^2+^ channels, or Gq-type GPCRs which elevate cytosolic Ca^2+^, as shown in human H295R adrenoma cells [52]. However, the relatively weak correlation between modulation of bio-impedance and Ca^2+^ mobilization (Table 1), potentially reflects uncoupling between Ca^2+^ signaling and changes in cell membrane properties with some venom fractions. The xCELLigence platform utilized disposable E-plates which contain a high density of gold electrodes arranged within a microelectrode array. As the rHEK293 cells adhered to this plate and proliferated, the CI impedance measurement increased slowly over hours as the conductive pathway became increasingly restricted by the cells covering the microelectrodes, as described by the manufacturer (ACEA Biosciences, Inc). Within the timeframe of our experiments (shown to 25 min but recorded to several hours), the venom activity was reversible. This contrasts with other published applications where cytotoxic effects on cell lines have been quantified using this platform as irreversible reductions in impedance as cells died and the electrode array was exposed. An example of where this assay has been used to screen toxin activity was with the Australian box jellyfish *Chironex fleckeri*, where the xCELLigence showed irreversible decreases in impedance resulting from cytotoxicity on human cardiomyocytes and muscle cells following exposure to crude toxin fractions; an effect observed within the timeframe of the present study [54]. Conversely, the extensive confocal imaging performed here demonstrated the stability of rHEK293 cell morphology (Figure 5), making it likely that *H. waigiensis* venom-induced changes in xCELLigence-measured impedance largely reflect modulation of the conductance across the cell monolayer due to changes biophysical properties. While the imaging of the cells was not at sufficient resolution to resolve direct effects on the cell membrane that would be detected as bio-impedance changes, gross changes in cell shape and number were not overtly altered.

The few peptides from other scorpion species previously shown to target cell voltage-gated Ca^2+^ channels (Cav) were all inhibitory. For example, kurtoxin (*Parabuthus transvaalicus*) selectively inhibited rat Cav3.1 and human Cav3.2 by raising the activation threshold (Kd 15 nM and 61 nM respectively) in the *Xenopus* expression system [55]. Additionally, α-KTx 2.15 and α-KTx 2.16 (*Centruroides tecomanus*) inhibit human Cav3.1 channels in tsA201-HEK293 cells [56]. In the Indian red scorpion *Mesobuthus tamulus*, only 0.8% of the toxin elements were identified as Ca^2+^ channel toxins, compared with the Na^+^ and K^+^ channel toxins which were most abundant at 76.7% [3]. This makes the increase in cytosolic Ca^2+^ elicited here by many of the *H. waigiensis* venom components likely to have arisen from intracellular Ca^2+^ store release. There is also a distinct possibility that *H. waigiensis* venom components, as for peptides in other scorpion species, also modulate Na^+^ and K^+^ channels, typically leading to membrane depolarization (for reviews [1,26,40]) and driving secondary activation of Cav channels. Given the complexity of the xCELLigence impedance responses, second and third order channel activation could contribute to the observed venom activity. 

While for drug discovery, the mode of action needs to be resolved at the level of individual isolated peptides, the current study clearly highlights the need to consider the interactions between activities of the bioactive molecules, with respect to the net pathophysiological outcome of envenomation. The GCaMP5G‒RyR1‒HEK293 bioreporter cell line had enhanced sensitivity for Ca^2+^ mobilization. While it was not possible to specifically delineate scorpion venom-mediated positive modulation of RyR channels, such action has previously been reported with calcin-like molecules isolated from several scorpion venoms [26]. In support of this, the temporal response for Ca^2+^ increases quickly in the present study (commencing within 30 sec of venom application and developing over 5–10 min), which closely matches data reported for the calcin imperacalcin (IpTxa; 3.7 kDa), when applied at 20 nM within a sarcoplasmic reticulum heavy vesicle model using the Ca^2+^ reporter Arsenazio III [41]. Additionally, in a cardiomyocyte-based Ca^2+^ imaging study using the fluorophore Fluo-3, IpTxa (100–300 nM) stimulated Ca^2+^ transients within seconds of application [43]. Including IpTxa, eight calcins are currently recognized [41], while overall 12 SCTX peptides are known to affect RyRs (reviewed by [26]), including the ϕ-liotoxin-Lw1a peptide from the same Australian scorpion used in the present study (*H. waigiensis*). 

ϕ-liotoxin-Lw1a shares structural homology with the calcin family and has been demonstrated to induce prolonged reversible subconductance states in RyR channels in vesiculated sheep cardiac sarcoplasmic reticulum and rabbit skeletal sarcoplasmic reticulum in lipid bilayers, inducing Ca^2+^ store release [47]. Indeed, the 4.171 kDa ϕ-liotoxin-Lw1a peptide has the highest potency (fM) demonstrated on RyR channels to date [47,57]. While our analysis incorporated the pooled venom from 14 *H. waigiensis* scorpions, LCMS did not identify a molecule with the molecular weight of the ϕ-liotoxin-Lw1a peptide. Our analysis of the size distribution of *H. waigiensis* venom components suggests that ϕ-liotoxin-Lw1a peptide would be within peak sample 2b–peak sample 6 (Figure 1D, Table S1). Given the potency of the ϕ-liotoxin-Lw1a peptide, and despite dilution through SE-FPLC fractionation and Ca^2+^ imaging sample preparation (~2100 and ~10,000-fold dilution respectively), both the xCELLigence platform and confocal GCaMP5G fluorescence assay would likely have been able to detect the ϕ-liotoxin-Lw1a peptide–mediated RyR activation. On this basis, it is possible that the ϕ-liotoxin-Lw1a peptide was indeed absent, or at un-detectible levels, in our venom samples, possibly reflecting the reported influence of environmental context on toxin proteomic plasticity, as shown in *H. waigiensis* [58]. 

While the identification of specific channel targets was precluded in the current study by the limited quantity of available venom, in the future, isolation of the individual SCTX peptides and small molecules within the peaks evident from our LCMS analysis will enable ion channel actions and Ca^2+^ mobilization mechanisms to be resolved.

## 5. Conclusions

The present study identified an unexpectedly broad bioactivity within the components of *H. waigiensis* scorpion venom that modulated cell layer impedance (as measured by the high-throughput real-time cell analysis xCELLigence platform) and raised cytosolic Ca^2+^ within a specifically constructed HEK293 cell line expressing the GCaMP5G fluorescent Ca^2+^ reporter and IP_3_R and RyR1 Ca^2+^ store-gated Ca^2+^ channels. The correlation of venom fraction bioactivity between bio-impedance and Ca^2+^ imaging platforms, despite differences in rank order potency, suggests xCELLigence can be reasonably used to screen bioactive compounds within scorpion venoms in future experiments. Our results demonstrate that the venom from this scorpion species will enable biodiscovery of peptides mobilizing cytosolic Ca^2+^. In particular, the study highlights the bioactivity of the smaller molecular weight molecules (<1 kDa), which fall outside the typical size of the channel blocker toxins, and are shown here to have significant potency for intracellular Ca^2+^ mobilization. Such biodiscovery strategies around scorpion venom components has tremendous potential for facilitating the development of novel pharmaceutical agents and therapeutics.

## Figures and Tables

**Table 1:** Comparison of rank orders of potency between the reduction in bioimpedance evident five minutes after application of Australian scorpion *Hormurus waigiensis* venom fractions and the Ca^2+^ response in GCaMP5G-RyR1-HEK293 cells.

Rank Order	xCELLigence-Based Bio-Impedance for Venom Pools	Mean Cell Index (Normalised Background-Subtracted)	Toxin-Induced Ca^2+^ Response for Venom Pool Equivalents	Sum of F/F_0_ Peak Samples *
**1**	**Pool B** **(peak samples 3 ‒4)**	‒0.199	**Pool D** **(peak samples 8‒10)**	3.114
**2**	**Pool C** **(peak samples 5‒7)**	‒0.153	**Pool C** **(peak samples 5‒7)**	3.105
**3**	**Pool A** **(peak samples 1‒2a,b)**	‒0.114	**Pool A** **(peak samples 1‒2a,b)**	3.048
**4**	**Pool E** **(peak sample 11)**	‒0.073	**Pool F** **(peak samples 12a,b)**	2.074
**5**	**Pool F** **(peak samples 12a,b)**	‒0.045	**Pool B** **(peak samples 3‒4)**	2.056
**6**	**Pool D** **(peak samples 8‒10)**	−0.020	**Pool E** **(peak sample 11)**	1.053

* the sum of F/F_0_ Ca^2+^ responses from individual venom peaks was used to (virtually) combine the Ca^2+^ signal component activity which may act on bio-impedance.

## Supplementary Materials

The following are available online at https://www.mdpi.com/2218-273X/10/4/617/s1, and includes the following references: [29,43,48,49,50], sections, figures, and tables: Development of the GCaMP5G-RyR1-HEK293 cell line, including Figure S1. Table S1. LCMS molecular weight determinations for SEC-FPLC peaks of pooled Australian Scorpion Hormurus waigiensis venom. Table S2. Size-ranked LCMS molecular weight distribution of Australian Scorpion *Hormurus waigiensis* venom. S3. Statistical analysis of xCELLigence bioimpedance measurements related to temporal profile in rHEK293-RyR1-GCaMP5G cells in response to application of SCTX fractions. Table S4. Comparison of variation in pooled venom-induced bioimpedance responses at 2.5 min in rHEK293-RyR1-GCaMP5G cells Table S5. Comparison of variation in pooled venom-induced bioimpedance responses at 5 min in rHEK293-RyR1-GCaMP5G cells. Table S6. Comparison of variation in pooled venom-induced bioimpedance responses at 25 min in rHEK293-RyR1-GCaMP5G cells. Table S7. Summary of *Hormurus waigiensis* venom-induced increases in cytosolic Ca2+.
