# Supplementary material for: Australian Scorpion Hormurus waigiensis Venom Fractions Show Broad Bioactivity through Modulation of Bio-Impedance and Cytosolic Calcium
Source: Biomolecules. 2020 Apr 16;10(4):617. doi: 10.3390/biom10040617 (PMC7226344; doi:10.3390/biom10040617)
Supplement: Supplementary file 1 [file biomolecules-10-00617-s001.pdf]

SUPPLEMENTARY MATERIALS

Article

Australian Scorpion *Hormurus waigiensis* Venom Fractions Show Broad Bioactivity Through Modulation of Bio-impedance and Cytosolic Calcium

David M. Housley[1,2,3], Jeremy L. Pinyon[1], Georg von Jonquieres[1], Chamini J. Parera[1], Michael Smout[4,5], Michael J. Liddell[6], Ernest A. Jennings[3,4,5], David Wilson[4,5] and Gary D. Housley[1*]

[1]Translational Neuroscience Facility and Department of Physiology, School of Medical Sciences, UNSW Sydney, Sydney, NSW 2052, Australia
[2]Department of Otolaryngology, Sunshine Coast University Hospital, Sunshine Coast, Queensland 4575
[3]College of Medicine and Dentistry
[4]Australian Institute of Tropical Health and Medicine
[5]Centre for Molecular Therapeutics
[6]Centre for Tropical Environmental and Sustainability Science, College of Science & Engineering, Cairns Campus, James Cook University, Cairns, Queensland 4878, Australia.

* Correspondence: g.housley@unsw.edu.au; Tel.: +61-2-93851057

INCLUDES:

- Development of the GCaMP5G-RyR1-HEK293 cell line, with Figure S1.
- Table S1. LCMS molecular weight determinations for SE-FPLC peaks of pooled Australian Scorpion *Hormurus waigiensis* venom
- Table S2. Size-ranked LCMS molecular weight distribution of Australian Scorpion *Hormurus waigiensis* venom
- Table S3. Statistical analysis of xCELLigence bioimpedance measurements related to temporal profile in rHEK293-RyR1-GCaMP5G cells in response to application of SCTX fractions
- Table S4. Comparison of variation in pooled venom-induced bioimpedance responses at 2.5 mins in rHEK293-RyR1-GCaMP5G cells
- Table S5. Comparison of variation in pooled venom-induced bioimpedance responses at 5 mins in rHEK293-RyR1-GCaMP5G cells
- Table S6. Comparison of variation in pooled venom-induced bioimpedance responses at 25 mins in rHEK293-RyR1-GCaMP5G cells
- Table S7. Summary of *Hormurus waigiensis* venom-induced increases in cytosolic Ca^{2+}

Development of the GCaMP5G-RyR1-HEK293 cell line

The GCaMP5F-RyR1-HEK293 recombinant human embryonic kidney 293 (rHEK293) biosensor cell line was established by selection for stable transfection with the genetically encoded Ca^{2+} reporter GCaMP5G, and the RyR1 type ryanodine receptor. The GCaMP5G genetically encoded Ca^{2+} reporter was a gift from Douglas Kim & Loren Looger; Addgene plasmid #31788) [1]. The rabbit RyR1 Ca^{2+}-store channel plasmid was provided by Dr. Paul Allen, University of California Davis, and Angela Dulhunty, Australian National University) (International Nucleotide Sequence Database Collaboration number: NM_001101718, GenBank). The RyR1 cDNA was cloned into the pCI-neo mammalian expression vector (Promega), which includes the human cytomegalovirus (CMV) immediate-early enhancer / promoter and the neomycin phosphotransferase gene, to permit selection for stable transfection using the antibiotic G418. The GCaMP5G plasmid was cloned into the p106 mammalian expression vector (generated by Dr Alexander Muravlev and provided by Dr. Matthias Klugmann) which included the CMV / chicken β-actin hybrid (CBA) promotor, and the woodchuck hepatitis virus posttranscriptional regulatory element (WPRE). These plasmids were used to transform competent *E. coli* cells (TOP10, Thermo Fisher Scientific). The plasmids were purified using minipreps (Qiagen Maxi prep) and validated via restriction digest. Adherent HEK293 cells (ThermoFisher Scientific) were maintained on plates using Dulbecco's Modified Eagle Medium (DMEM) media, supplemented with 10% fetal calf serum (Gibco, USA), in a humidified cell culture incubator (37 ^{o}C, 5% CO_2). The RyR1 plasmid and the GCaMP5 plasmid were linearized via Acl1 and Msc1 restriction enzymes respectively, and then transfected into the HEK293 cell line via electroporation.

Integration of the RyR1 plasmid was selected for via application of G418 antibiotic, during cell passage, over a 4-month period to ensure the establishment and maintenance of the stably transfected cell line (600 µg / ml for selection and 200 µg / ml for maintenance). This was undertaken alongside a control non-transfected HEK293 cell line, which did not survive this selection, validating expression of the RyR1 plasmid. HEK293 cells lack significant intrinsic expression of RyRs [2]. The dual expression of RyR1 and GCaMP5G plasmids within the G418-selected HEK293 cells was validated by recording an increase in fluorescence in response to the application of 4 mM caffeine (RyR agonist) [3], measured using a confocal laser scanning microscope (see below). Fluorescence Activated Cell Sorting (FACS) was then performed to select for the top 25% of baseline (GCaMP5G) fluorescence. This mixed cell line (rHEK293) was then cryopreserved at -80 ^{o}C in DMEM containing 10% DMSO.

To validate the RyR1-based Ca^{2+} store release, caffeine-induced Ca^{2+} dynamics were measured using the GCaMP5G fluorescence of rHEK293-RyR1-GCaMP5G cells in comparison to HEK293-GCaMP5G cells (lacking RyR1 expression), using a FlexStation 3 multi-mode microplate reader (Molecular Devices, CA, USA) (Figure S1). The DMEM media in the wells was substituted with a physiological saline solution (in mM: NaCl 145; KCl 5.8; $MgCl_2$ 0.9; $CaCl_2$ 1.3; HEPES 10, NaH_2PO_4 0.7, D-glucose 5.6; pH 7.2 – 7.4, osmolarity 310 – 330 mOsm/L) just prior to Ca^{2+} imaging (> 95% cell confluence). The 'Flex Read' configuration was utilized (485 nm excitation, 510 nm emission, 6 flashes per sample, medium detector sensitivity, 37 ^{o}C, 585 sample points at 1.54s intervals). Caffeine (Sigma) solution (10 µl) was added at 20x target concentration for a final volume of 200 µl using the robotic dispenser.

The dual RyR1 and Ca^{2+} reporter cell line was functionally validated using fluorescence spectroscopy, comparing the peak Ca^{2+} signal associated with release of Ca^{2+} from endoplasmic reticulum with application of caffeine (40 µM - 4 mM) in wells of rHEK293-RyR1-GCaMP5G cells, against comparable wells of rHEK293-GCaMP5G cells (Figure S1). The dose response data (Figure S1B) showed that over-expression of the RyR1 channels had a 4.5 fold greater change in fluorescence signal from (4 mM) caffeine-mediated Ca^{2+} store release ($p < 0.0001$; two way ANOVA). The existence of a weak caffeine response in the rHEK293-GCaMP5G cells lacking the recombinant RyR1 expression cassette is consistent with variable endogenous RyR1 and RyR2 expression [2,4,5].

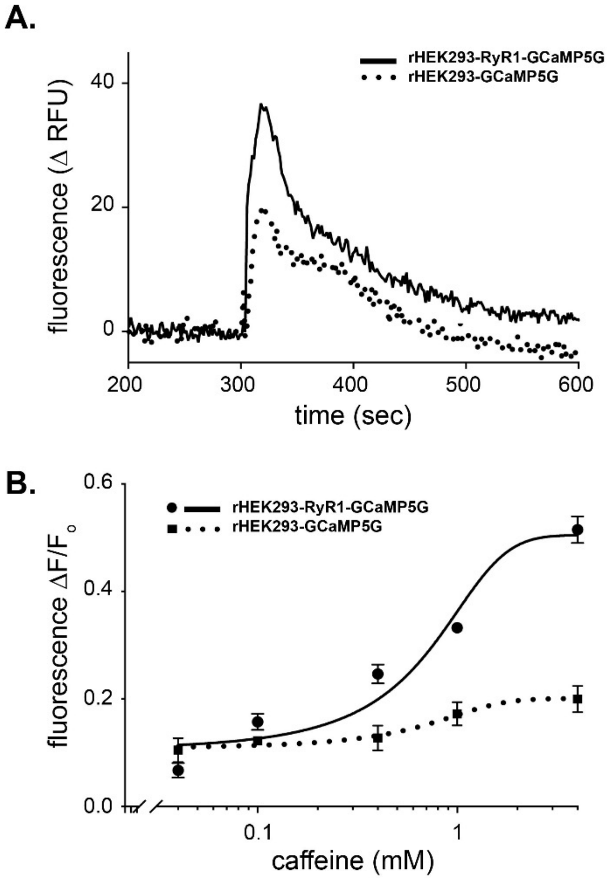

Figure S1. Enhanced ryanodine receptor (RyR) – mediated Ca^{2+} store activation in GCaMP5G-RyR1-HEK293 cells. Comparison of caffeine-induced cytosolic Ca^{2+} signals in HEK293 cells expressing the GCaMP5G genetically encoded Ca^{2+} reporter with (GCaMP5G-RyR1-rHEK293), and without (rHEK293-GCaMP5G), co-expression of the recombinant rabbit RyR1 receptor (rRyR1), recorded using a FlexStation 3 multi-mode fluorescence spectroscopy microplate reader. (A) Fluorescence based Ca^{2+} responses from individual wells to 4 mM caffeine, added at the 300 second time point (190 µl starting volume in imaging media, 10 µl x 80 mM caffeine-containing media added; ΔRFU – change in relative fluorescence units). (B) Dose response curves comparing the rRyR1 expressing cell line against the cell line lacking rRyR1 expression shown as ΔF/F0 (peak change in fluorescence normalized to baseline fluorescence). The EC50 is ~ 1 mM for both cell lines. The 4 mM caffeine response of the rRyR1 expressing cell line was 0.40 ΔF/F0, compared with 0.09 ΔF/F0 for the endogenous response. N = 4 wells per data point (mean ± s.e.m.).

Table S1.

LCMS molecular weight determinations for SE FPLC peaks of pooled Australian Scorpion *Hormurus waigiensis* venom (Da)

Peak	Peak 1	Peak 2a	Peak 2b	Peak 3	Peak 4	Peak 5	Peak 6	Peak 7	Peak 8	Peak 9	Peak 10	Peak 11	Peak 12a	Peak 12b
Elution Fraction	1-5	6-8	9-11	12-14	15-18	19-22	23-24	25-26	27-29	30-32	33-36	37-43	44-48	49-53
no. molecules	4	5	23	26	48	63	36	31	28	18	13	16	2	2
	7921.2250	7905.9475	1785.5484	1101.4344	409.6435	265.4444	262.1531	258.0588	262.1386	278.0147	277.0265	262.0856	408.0020	350.9934
	7945.4004	7920.7543	2997.2090	1229.5953	446.3271	279.1181	275.8701	262.1310	275.8106	283.0938	278.0213	275.7995	465.0417	465.0464
	9257.9274	7946.2041	3754.9672	2997.5620	614.4969	315.0385	335.0239	275.8032	283.0990	297.9851	297.9889	277.0446		
	11846.2411	8572.7158	4983.1984	3672.2236	904.3029	338.1963	352.2272	297.9897	297.9809	303.0462	303.0486	296.6007		
		9257.9444	5431.3821	3754.9631	1101.4344	351.9072	357.6924	335.0397	317.0459	314.9770	314.9757	297.9780		
			5605.5539	3900.2594	1169.5950	359.8793	365.0319	343.0456	335.0391	319.0457	317.1177	317.1572		
			7198.5813	4377.7972	1600.9972	365.8547	374.0440	344.8700	343.0260	343.0633	319.0480	343.0493		
			7906.4745	4572.6596	2997.4991	400.6887	404.0449	365.0324	365.0336	355.9960	343.0588	360.9821		
			7920.3703	4983.7111	3013.1250	403.5924	408.0139	374.0462	394.7159	360.0156	356.0172	372.6965		
			7946.5891	5430.7141	3016.0531	411.2468	417.0656	408.0308	404.0569	394.6612	465.0382	408.0504		
			8248.0332	6358.6720	3089.2708	427.6544	430.0113	417.0435	407.7310	431.0586	525.1810	438.0333		
			8263.8477	6371.6173	3100.2950	432.0870	431.0580	429.9704	408.0513	447.0540	554.2611	447.0364		
			8411.0650	7198.7855	3146.3432	452.0855	446.0656	431.0657	431.0532	525.1010	817.3025	465.0396		
			8573.0291	7785.7290	3171.2539	486.7929	505.1333	439.3063	446.0793	544.1718		593.1292		
			9180.7695	7906.5301	3270.5420	515.2609	522.1639	446.0686	447.0712	554.1811		633.2516		
			9257.4578	7920.6847	3284.3621	522.0133	562.1518	448.2626	448.2791	618.1328		817.3025		
			9344.0230	7946.5307	3286.6203	530.1215	569.1991	473.0952	465.0647	691.2657				
			11251.1077	8247.9183	3289.5750	540.1811	614.2614	505.1167	488.1009	817.2224				
			12323.1462	8410.6332	3342.2705	544.3011	648.2546	516.1693	505.1137					
			12471.1785	8572.2425	3486.6214	592.2047	664.8050	522.1542	525.1010					
			12575.9427	9180.9909	3672.0856	595.9324	674.1891	544.1847	544.1957					
			13558.9128	9257.6778	3690.0612	614.3015	704.2219	562.1545	545.1506					
			13695.4382	9344.1691	3852.0521	704.1019	745.2221	569.1481	562.1471					
				12575.8672	3874.8399	796.2410	789.3024	573.2212	573.3012					
				12784.9447	3890.3714	817.3025	815.2434	618.1136	618.1028					
				13695.5615	3900.3284	822.3025	817.3025	703.1838	627.1854					
					3970.5613	850.3026	843.2227	722.4085	745.3000					
					3978.6533	904.3429	930.3030	745.3021	817.3025					
					3996.1909	915.3430	1201.4245	815.3025						
					4043.2583	1002.4635	1289.7340	817.2224						
					4206.0236	1038.4708	2698.2994	843.2738						
					4469.6256	1051.4237	3852.4299							
					4491.8946	1056.4342	3978.3287							
					4572.5734	1110.5440	3996.8037							
					4655.9639	1169.7334	7605.1791							
					5430.2391	1201.3445	7704.6606							
					6358.4569	1228.7645								
					6371.6786	1282.7603								
					6451.0539	1289.7340								
					6975.9939	1314.4651								
					7785.6514	1401.0578								
					8410.8059	1601.1356								
					9145.4501	1934.5408								
					9257.6664	2102.4824								
					9344.3435	2698.2814								
					11846.3620	2878.5989								
					12786.2183	3016.0531								
					13695.2610	3284.2340								
						3286.6752								
						3342.4775								
						3672.0166								
						3673.7549								
						3793.8527								
						3851.5014								
						3875.6289								
						3909.2766								
						3970.0414								
						3978.0871								
						3996.1563								
						4043.1037								
						4152.4652								
						4763.1841								
						6975.8901								

Table S2.

Size-ranked LCMS molecular weight distribution of Australian Scorpion *Hormurus waigiensis* venom (Da)

1	258.0588	262.0856	262.1531	265.4444	275.8701	275.7995	277.0265	278.0147	279.1181	283.0938	296.6007	297.978	298.958	303.0462	314.9757	315.0385	317.1177	317.0459	319.0457	
20	335.0239	338.1963	343.026	344.87	350.9934	351.9072	356.0172	357.6924	359.8793	360.0156	360.9821	365.0319	372.6965	374.044	394.6612	400.6887	403.5924	408.002	409.6435	411.2468
40	417.0435	427.6544	430.0113	431.0532	432.087	438.0333	439.3063	446.0656	446.3271	447.054	448.2626	452.0855	465.0382	473.0952	486.7929	488.1009	505.1137	515.2609	516.1693	522.0133
60	525.101	530.1215	540.1811	544.3011	544.1718	545.1506	554.1811	562.1471	569.1481	573.2212	592.2047	593.1292	595.9324	614.2614	618.1028	627.1854	633.2516	648.2546	664.805	674.1891
80	674.5726	691.2657	703.1838	704.1019	722.4085	745.2221	789.3024	796.241	815.2434	817.2224	822.3025	843.2227	850.3026	904.3029	915.343	930.303	1002.4635	1038.4708	1051.4237	1056.4342
100	1101.4344	1110.544	1169.595	1201.3445	1228.7645	1229.5953	1282.7603	1289.734	1314.4651	1401.0578	1600.9972	1785.5484	1934.5408	2102.4824	2698.2814	2878.5989	2997.209	3013.125	3016.0531	3089.2708
120	3100.295	3146.3432	3171.2539	3284.234	3286.6203	3289.575	3342.2705	3342.4048	3486.6214	3672.0166	3673.7549	3690.0612	3754.9631	3793.8527	3851.5014	3875.6289	3890.3714	3900.2594	3909.2766	3970.0414
140	3978.1907	3996.1563	4043.1037	4152.4652	4206.0236	4377.7972	4469.6256	4492.2394	4572.5734	4655.9639	4763.1841	4983.1984	5430.7141	5605.5539	6358.4569	6371.6173	6451.0539	6975.8901	7198.5813	7605.1791
160	7704.6606	7785.6514	7905.9475	7920.3703	7946.5307	8248.0332	8263.8477	8410.6332	8572.7158	9145.4501	9180.7695	9257.6664	9344.1691	11251.1077	11846.3620	11846.8034	12323.1462	12471.1785	12575.9427	12784.9447
180	12786.2183	13558.9128	13695.4382																	

Table S3. Statistical analysis of xCELLigence bioimpedance measurements related to temporal profile in rHEK293-RyR1-GCaMP5G cells in response to application of SCTX fractions

Group	Mean CI* ± s.e.m.	N	t stat	*P* (one sample t-test, two tailed)	*P* < 0.05
Whole Venom (2.5 min)	0.057 ± 0.018	6	3.218	0.0235	Yes
Whole Venom (5 min)	-0.194 ± 0.029	6	6.771	0.00107	Yes
Whole Venom (25 min)	0.104 ± 0.037	6	2.782	0.0388	Yes
Reconstituted Venom (2.5 min)	0.062 ± 0.008	6	7.852	0.000538	Yes
Reconstituted Venom (5 min)	-0.139 ± 0.028	6	**Z = -2.201	0.031	Yes
Reconstituted Venom (25 min)	0.072 ± 0.037	6	**Z = 1.153	0.313	No
Pool A (2.5 min)	-0.078 ± 0.017	8	-4.632	0.00239	Yes
Pool A (5 min)	-0.114 ± 0.008	8	-13.595	<0.0001	Yes
Pool A (25 min)	0.046 ± 0.016	8	2.883	0.0236	Yes
Pool B (2.5 min)	-0.152 ± 0.022	9	-6.920	0.000122	Yes
Pool B (5 min)	-0.199 ± 0.020	9	**Z= -2.666	0.004	Yes
Pool B (25 min)	0.008 ± 0.034	9	**Z= 0.415	0.734	No
Pool C (2.5 min)	-0.070 ± 0.022	7	-3.241	0.0177	Yes
Pool C (5 min)	-0.153 ± 0.017	7	-8.929	0.00011	Yes
Pool C (25 min)	0.101 ± 0.019	7	**Z= 2.197	0.031	Yes
Pool D (2.5 min)	-0.025 ± 0.005	5	-5.555	0.00514	Yes
Pool D (5 min)	-0.020 ± 0.004	5	-5.278	0.00618	Yes
Pool D (25 min)	0.015 ± 0.007	5	2.101	0.103	No
Pool E (2.5 min)	0.061 ± 0.012	6	4.908	0.00444	Yes
Pool E (5 min)	-0.073 ± 0.014	6	-5.342	0.00308	Yes
Pool E (25 min)	-0.015 ± 0.013	6	-1.121	0.313	No
Pool F (2.5 min)	-0.060 ± 0.026	7	-2.153	0.0748	No
Pool F (5 min)	-0.045 ± 0.023	7	**Z= -2.366	0.016	Yes
Pool F (25 min)	0.045 ± 0.029	7	**Z= 1.690	0.109	Yes

* Normalized background subtracted CI (cell index) data

** One-sample Signed Rank Test

Table S4. Comparison of variation in pooled venom-induced bioimpedance responses at 2.5 mins in rHEK293-RyR1-GCaMP5G cells

Comparison	Difference of CI means*	t stat	*P* (ANOVA)	*P* < 0.05
E vs. B	0.213	7.314	<0.001	Yes
E vs. A	0.139	4.666	<0.001	Yes
E vs. C	0.132	4.280	0.001	Yes
D vs. B	0.127	4.113	0.002	Yes
E vs. F	0.118	3.826	0.004	Yes
F vs. B	0.095	3.425	0.012	Yes
C vs. B	0.081	2.924	0.046	Yes

* Normalized background subtracted CI (cell index) data

Table S5. Comparison of variation in pooled venom-induced bioimpedance responses at 5 mins in rHEK293-RyR1-GCaMP5G cells

Comparison	Difference of CI means*	t stat	*P* (ANOVA)	*P* < 0.05
D vs. B	0.179	5.823	<0.001	Yes
F vs. B	0.155	5.561	<0.001	Yes
E vs. B	0.127	4.358	<0.001	Yes
D vs. C	0.133	4.118	0.002	Yes
F vs. C	0.109	3.676	0.006	Yes
A vs. B	0.085	3.173	0.026	Yes
D vs. A	0.094	2.993	0.038	Yes

* Normalized background subtracted CI (cell index) data

Table S6. Comparison of variation in pooled venom-induced bioimpedance responses at 25 mins in rHEK293-RyR1-GCaMP5G cells

Comparison	Difference of CI means*	t stat	*P* (ANOVA)	*P* < 0.05
C vs. E	0.116	3.785	0.006	Yes
C vs. B	0.093	3.340	0.022	Yes

* Normalized background subtracted CI (cell index) data

Table S7. Summary of *Hormurus waigiensis* venom-induced increases in cytosolic Ca^{2+}

Treatment Group	Mean F/F_0 response ± s.e.m..	Number	t Stat*	*P* value	Significance (α<0.05)
**Whole venom**	1.073 ± 0.001	3	54.246	0.00017	Yes
**Peak sample 1**	1.047 ± 0.010	3	4.875	0.0198	Yes
**Peak sample 2a**	1.020 ± 0.010	3	2.099	0.0853	No
**Peak sample 2b**	1.017 ± 0.001	3	12.895	0.00298	Yes
**Peak sample 3**	1.028 ± 0.008	3	3.400	0.0383	Yes
**Peak sample 4**	1.028 ± 0.008	3	3.625	0.0342	Yes
**Peak sample 5**	1.024 ± 0.001	3	16.987	0.00172	Yes
**Peak sample 6**	1.044 ± 0.020	3	2.165	0.0814	No
**Peak sample 7**	1.037 ± 0.011	3	3.379	0.0388	Yes
**Peak sample 8**	1.027 ± 0.016	3	**Z = 1.604	0.250	No
**Peak sample 9**	1.047 ± 0.017	3	2.726	0.0562	No
**Peak sample 10**	1.040 ± 0.011	3	3.736	0.0324	Yes
**Peak sample 11**	1.053 ± 0.001	3	59.354	0.000142	Yes
**Peak sample 12a**	1.053 ± 0.003	3	10.446	0.00452	Yes
**Peak sample 12b**	1.038 ± 0.020	3	1.935	0.0963	No
**Control (PBS & media)**	1.003 ± 0.005	5	0.677	0.268	No
**Caffeine**	1.070 ± 0.006	44	12.130	< 0.0001	Yes

*single sample t-tests, one-tailed, testing mean = 1

**one-sample Signed Rank Test

Supplementary Materials References

1. Akerboom, J.; Chen, T.W.; Wardill, T.J.; Tian, L.; Marvin, J.S.; Mutlu, S.; Calderon, N.C.; Esposti, F.; Borghuis, B.G.; Sun, X.R., et al. Optimization of a GCaMP calcium indicator for neural activity imaging. *J Neurosci* **2012**, *32*, 13819-13840, doi:10.1523/JNEUROSCI.2601-12.2012.
2. Tong, J.; Du, G.G.; Chen, S.R.; MacLennan, D.H. HEK-293 cells possess a carbachol- and thapsigargin-sensitive intracellular Ca2+ store that is responsive to stop-flow medium changes and insensitive to caffeine and ryanodine. *Biochem J* **1999**, *343 Pt 1*, 39-44.
3. Gurrola, G.B.; Capes, E.M.; Zamudio, F.Z.; Possani, L.D.; Valdivia, H.H. Imperatoxin A, a Cell-Penetrating Peptide from Scorpion Venom, as a Probe of Ca(2+)-Release Channels/Ryanodine Receptors. *Pharmaceuticals (Basel)* **2010**, *3*, 1093-1107, doi:10.3390/ph3041093.
4. Querfurth, H.W.; Haughey, N.J.; Greenway, S.C.; Yacono, P.W.; Golan, D.E.; Geiger, J.D. Expression of ryanodine receptors in human embryonic kidney (HEK293) cells. *Biochem J* **1998**, *334 (Pt 1)*, 79-86, doi:10.1042/bj3340079.
5. Luo, D.; Sun, H.; Xiao, R.P.; Han, Q. Caffeine induced Ca2+ release and capacitative Ca2+ entry in human embryonic kidney (HEK293) cells. *Eur J Pharmacol* **2005**, *509*, 109-115, doi:10.1016/j.ejphar.2004.12.038.
